# Mechanical Properties and Microanalytical Study of Concrete Reinforced with Blended Corn Straw and Scrap Steel Fibers

**DOI:** 10.3390/ma17153844

**Published:** 2024-08-02

**Authors:** Jingjing He, Chuanwu Sun, Xuezhi Wang

**Affiliations:** 1 PowerChina Northwest Engineering Corporation Limited, Xi’an 710065, China; hejing_86@126.com; 2 School of Civil and Architectural Engineering, Liaoning University of Technology, Jinzhou 121001, China

**Keywords:** corn straw fiber, scrap steel fiber, fiber concrete, basic mechanical properties, impact strength, hierarchical analysis method

## Abstract

Fiber concrete exhibits superior performance in various aspects compared to plain concrete and has been widely researched and applied worldwide. However, many industrially made fibers are expensive, and their cost has to be considered before use; thus, it would be economically valuable to find inexpensive fibers with excellent properties to make fiber concrete. Rural areas have many rich straw resources to be disposed of; at the same time, the rapid development of the automobile industry has introduced a large number of used tires containing steel wire with a very low reuse rate. These two low-cost materials can be processed to make fibers, making the study of mechanical properties regarding their incorporation into concrete practically significant for reducing the cost of fiber concrete. Based on this, a three-factor, three-level orthogonal test was conducted to investigate the effects of different dosages of corn straw fibers and scrap steel fibers, as well as the water–cement ratio, on the mechanical properties of concrete. The optimum level of each factor for blended straw–waste-steel-fiber concrete with different mechanical properties was obtained using the polar and ANOVA methods. It was found that the compressive strength, splitting tensile strength, flexural strength, and impact resistance of the specimens after fiber dosing were better than those of plain concrete specimens with the same water–cement ratio. The maximum improvement was 14.96% in cubic compressive strength, 42.90% in tensile strength, and 16.30% in flexural strength, while the maximum improvement in impact energy consumption at the final crack was 228.03%. Combined with SEM microanalysis, the two fibers formed a stronger whole with the C-S-H gel. When the specimen was subjected to load, the two fibers were able to withstand part of the load, thus enhancing the load-bearing capacity. Finally, the optimal mix ratio of blended straw–scrap-steel-fiber concrete was determined to be 0.8% corn straw fibers by volume, 0.6% scrap steel fibers by volume, and a 0.45 water–cement ratio by combining the weights of the levels of each factor under its four different mechanical properties through hierarchical analysis. This analysis of mechanical properties provides a reference for practical applications in future projects.

## 1. Introduction

Ordinary concrete, with its strong compressive properties, also exhibits inherent low-tensile properties and poor toughness, prompting the development of fiber-reinforced concrete. This material is straightforward to fabricate and provides a degree of improvement in tensile strength, toughness, and durability [1,2,3]. Adding fibers to concrete can prevent crack propagation through a bridging effect formed by the fibers, and it also improves the durability of concrete [4,5]. Different types of fibers vary in their properties and enhance different aspects of concrete to varying degrees. However, some fibers are expensive and hold huge economic value.

In the past, the method used to treat straw was incineration, which produces harmful gasses that have a detrimental impact on the environment and human beings [6]. Many experts and scholars believe that if straw fibers, which are cheap to manufacture, are applied to the construction industry [7], it could not only broaden rural straw treatment methods but also reduce the production cost of fiber concrete [8,9]. Dušek et al. [10] used a water and sodium hydroxide solution to modify rapeseed straw, along with bone glue adhesive, to make an energy-efficient and sustainable composite building material. Their test found that water and sodium hydroxide pretreatment of the straw surface improved the interfacial adhesion between the products. Incorporating waste or inexpensive fibers that maintain these advantageous properties could create a stronger bond between the rapeseed straw and bone glue. Niyigena et al. [11] found that the particle size of Hanseatic straw has a significant effect on the mechanical properties of Hanseatic straw fiber concrete, with a smaller particle size of Hanseatic straw resulting in better concrete mechanical properties. Aldaood et al. [12] investigated the incorporation of different straw fiber and cement dosages into soil to make straw fiber-reinforced clay. They concluded that the incorporation of 0.5% of straw fibers increased the compressive strength of hydromodified soil the most, and it was also found that the toughness of the soil samples with the incorporation of straw fibers was also improved. Agwa et al. [13] investigated the use of rice and cotton straw ash to replace part of the cement needed to make concrete in order to make lightweight, self-compacting concrete. It was found that the compressive strength of concrete containing 5% and 10% cotton straw ash was improved relative to the control group, as was the flexural strength of the concrete containing 13%, 15%, and 22% cotton straw. Meanwhile, the lightweight self-compacting concrete made with 10% rice straw ash became more structurally dense. Bheel et al. [14] tested the compressive strength, modulus of elasticity, splitting tensile strength, and flexural strength of concrete made by replacing part of the cement with wheat straw ash. They found that the use of 10% wheat straw ash increased the compressive strength, splitting tensile strength, and flexural strength of the concrete by 12%, 10%, and 11%, respectively. Wang et al. [15] found that the smaller the diameter of the straw fibers, the better the thermal insulation of the concrete, and that the addition of straw fibers inside the concrete decreased its overall density, which is the main reason for the increase in thermal insulation. Toledo et al. [16] investigated the durability of fibers in an alkaline environment of cement and concluded that silica fume or blast-furnace slag can be used to replace part of the cement to reduce the alkalinity of the material and to improve the early carbonation of the composite material, which can effectively solve the problem of low durability that arises when using plant fibers in composites. The addition of straw fibers can increase the continuity of concrete, which in turn can improve the carbonation resistance of concrete.

Scholars in the United States were the first to propose the concept of steel fiber concrete last century, and since then, steel fiber concrete has been widely developed. Tire filaments in tires have properties similar to those of steel fibers [17,18,19]. Car ownership in China increases yearly, with a huge production of automobile tires, which typically have a service life of approximately five to six years [20]. According to the National Bureau of Statistics, China, being a major automobile country, generated 379.8 million waste tires, weighing 14.59 million tons, in 2018. With an annual growth rate of 6–8%, the amount of waste tires generated in 2020 officially surpassed the 20 million tons mark. Currently, the treatment of end-of-life tires mostly involves recycling the rubber part of the tires, and for most of the tires, the wire in the tires is remelted by the steel industry and seldom used directly for other purposes. Therefore, the steel wire fibers from scrap tires have great research prospects for application in fiber concrete. Scholars have found that these fibers have the potential to replace industrial steel fibers [21,22,23]. Ramakrishna et al. [24] conducted bending tests on plate members based on the research of previous scholars. Their results showed that the performance of scrap-steel-fiber concrete and industrial steel fiber concrete was comparable. Aiello [25] studied the irregular shape of scrap steel fibers, which was more conducive to the adhesion between the fibers and the concrete than industrial steel fibers, and the tensile properties were better. Antonio Caggiano [26] found that steel fibers extracted from scrap tires have promising applications and that industrial steel fibers can be replaced by an equal or slightly higher admixture of scrap steel fibers with the maintenance of their mechanical properties. Mastali et al. [27] investigated the mechanical properties and impact resistance of self-compacting concrete mixed with scrap steel fibers and industrial steel fibers. The results showed that the replacement of industrial steel fibers with scrap steel fibers had the greatest effect on the residual flexural strength, final cracking resistance, and densification. In addition, they found that the mixing of scrap steel fibers and industrial steel fibers had a greater effect on costs than on carbon emissions. Sengul [28] compared the similar mechanical properties of industrial and scrap steel fibers. Regarding the cost, the cost of the scrap steel fibers was lower. It was also found that the mechanical properties of used steel fibers do not deteriorate significantly and thus they can be used as a substitute for industrial steel fibers. Al-Tikrite A et al. [29] found that the flowability of activated powder concrete was significantly affected by the addition of scrap steel fibers recycled from waste tires at 4%. Maximum values of tensile strength, stress, and strain were obtained with a 4% addition of scrap steel fibers.

Based on the above considerations, if corn straw fibers and scrap steel fibers are blended to form green and environmentally friendly concrete, the application prospects of fiber concrete can be greatly enhanced. In this paper, scrap steel fibers were used as high-elastic-modulus fibers and corn straw fibers were used as low-elastic-modulus fibers to prepare a blended straw–scrap-steel-fiber concrete. A three-factor, three-level orthogonal test was designed to investigate the effects of different corn straw fiber dosages, waste steel fiber dosages, and water–cement ratios on the mechanical properties (compressive strength, splitting tensile strength, flexural strength, and impact strength) of the concrete. The significance of different factors and the optimal levels of various properties were analyzed using polar analysis and variance analysis, while the optimal mix ratio was determined using the comprehensive influence weights of hierarchical analysis. The results provide a certain theoretical basis for the promotion and application of green concrete in the future.

## 2. Experimental Design

### 2.1. Raw Material

**Figure 1:**
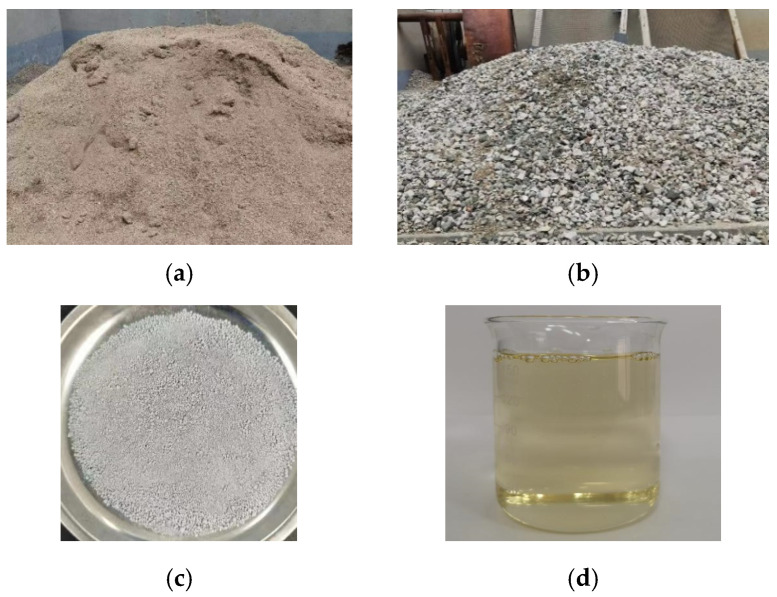
Raw materials. (**a**) River sand; (**b**) coarse aggregate; (**c**) cement; (**d**) water-reducing agent.

The fine aggregate was made from Jinzhou’s local river sand, with a fineness modulus of 2.41; its related parameters are shown in Table 1. The coarse aggregate was made of natural gravel with a continuous grain size of 5–20 mm; the physical indexes of this are shown in Table 2. The cement used in this experiment was 42.5-grade ordinary silicate cement, and the chemical composition of the cement is shown in Table 3. The water-reducing agent was a polycarboxylic acid water-reducing agent produced by Zhongyan Building Material Company, and the water-reducing rate was 15–30%. The appearance of the materials is shown in Figure 1.

The corn straw fibers were made from locally collected corn stalks from Jinzhou, and the scrap steel fibers were obtained from steel wire fibers separated from crushed scrap tires in local rubber factories in Jinzhou.

**Figure 2:**
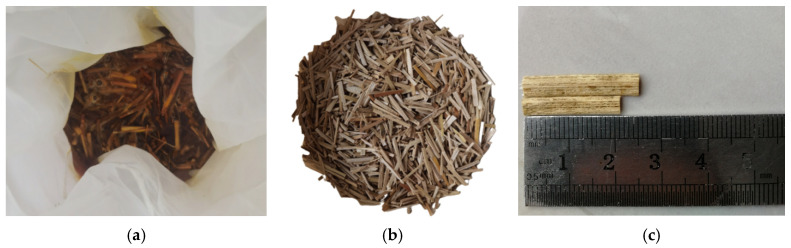
Corn straw fibers. (**a**) Soaking process; (**b**) after immersion; (**c**) length schematic.

Scholars at home and abroad use water immersion, alkali treatment, and hydrogen peroxide solution immersion [8] to remove harmful substances from straw fibers, change the roughness of the straw surface, and hydrolyze detrimental components such as hemicellulose. In this experiment, the corn straw fibers were soaked in a 4% NaOH solution for 24 h. After soaking, the straw was washed with water and placed in an open space to dry naturally. The reason for using alkali treatment was mainly because the compatibility between natural corn straw fibers and cement is not good. The surface hemicellulose and lignin of the straw affect the hydration reaction of the cement. Soaking can mitigate the negative effects of this material-and-alkali reaction and thus most of the detachment, leading to better adhesion of the straw fibers to the cement paste by roughening their surface [30]. The process of corn straw fiber treatment is shown in Figure 2, and the relevant parameters of the corn straw fibers are shown in Table 4.

**Figure 3:**
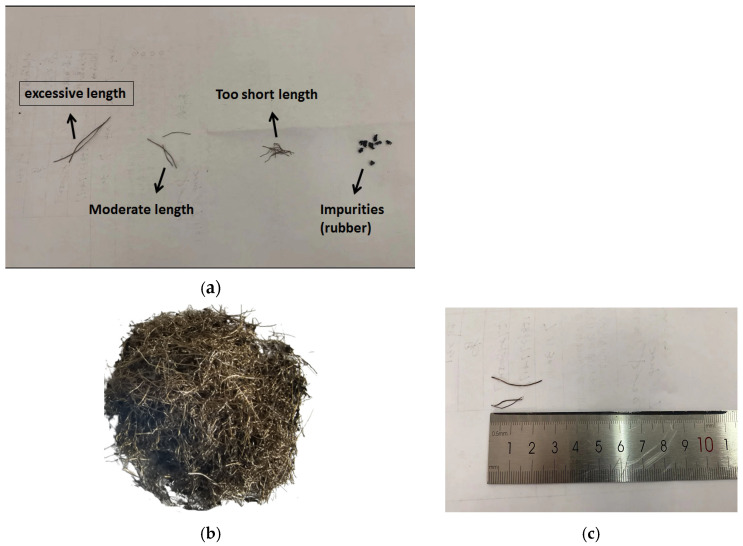
Scrap steel fibers. (**a**) Screen before sifting; (**b**) screen after sifting; (**c**) length schematic.

Steel wire fibers separated from automobile tires have different shapes and are broken to varying degrees, and they may contain impurities from the crushed tires. To ensure accurate test results, it is necessary to determine in advance the acceptable range of lengths for the waste steel fibers used in the test, considering that excessively long steel wires could potentially affect the outcomes. In order to ensure that our tests were not affected by extraneous variables, it was necessary to screen out the small amount of rubber particles in the fibers and any excessively long or short fibers using a sieve, leaving scrap wire fibers of approximately 1–3 cm in length. Since the fibers were found to be more prone to agglomeration during this sieving process, the use of sieves was also required for the later mixing of the scrap steel fibers into the concrete. Their appearance is shown in Figure 3, and the related parameters are shown in Table 5.

### 2.2. Test Equipment

Concrete preparation was carried out using a vertical mortar mixer model IW-400 manufactured by Beijing Lu Xing Group, Beijing, China. Mechanical tests for cubic compressive strength, split tensile strength, and flexural strength were conducted using a YAW-5000J compression shear testing machine (Dawson Group Co., Ltd., Qingdao, China). For the impact resistance test, a falling hammer impact tester manufactured by Liaoning University of Technology, China, was used in accordance with the method recommended by the American Concrete Institute.

### 2.3. Mixing Ratio Design

Based on the “Ordinary Concrete Proportion Design Regulations” (JGJ55-2011) [31], the different trial mixtures of concrete with strength C40 are detailed in Table 6. Given that this study required a large number of specimens, a three-factor, three-level orthogonal test was employed to effectively reduce the number of test groups. This design was chosen with the aim of studying the effects of varying volumes of corn straw fibers and scrap steel fibers on the mechanical properties of concrete. Additionally, due to the increased water absorption caused by the drying nature of the corn straw fibers, the water–cement ratio was also considered as a factor, as shown in Table 7.

### 2.4. Specimen Design

The test blocks used were designed and fabricated in accordance with the Standard for Test Methods of Mechanical Properties of Ordinary Concrete (GB50081-2002) [32], in which the cubic compressive strength was 100 × 100 × 100 mm, the splitting tensile strength was 100 × 100 × 100 mm, and the flexural strength was 100 × 100 × 400 mm. The impact strength specimens were made of round cake with a height of 63 mm and a diameter of 150 mm. The strength of the specimens was tested after 28 days of indoor curing under natural conditions. The number of specimens per group for compressive strength, splitting tensile strength, and flexural strength testing was 3, and the number of specimens per group for impact strength testing was 6.

### 2.5. Specimen Fabrication

**Figure 4:**
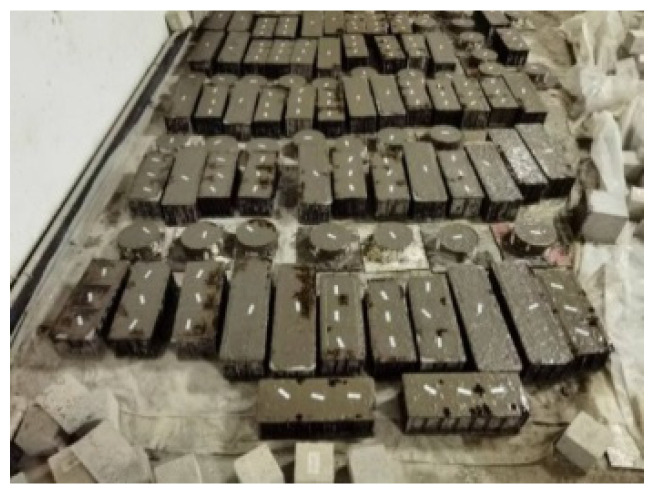
Specimen curing.

Based on specimen preparation using a forced concrete mixer for on-site mechanical mixing, the preparation process was strictly based on the relevant provisions of the Technical Specification for Fiber Concrete Application (JGJ/T221-2010) [33]. In order to prevent the phenomenon of agglomeration when two kinds of fibers are mixed in large amounts, after adding the fine aggregate, cement, and coarse aggregate, the corn straw fibers were added by hand and mixed dry for 3–5 min. In order to prevent the waste steel fibers from agglomerating and sinking to the bottom, after adding water and a water-reducing agent to the mix, the waste steel fibers were contained in a sieve, and the sieve was shaken manually at a uniform speed so that the waste steel fibers could be evenly dispersed into the mixed concrete. When the cement, aggregate, and fibers were fully mixed, they were loaded into the mold, and then put on the electric vibration table to undergo vibration, compaction, and molding. After 24 h of condensation molding, the specimens were removed from the mold and cured for 28 days, ready for data collection. In the conservation room, shown in Figure 4, the temperature conditions were 25 ± 2 °C.

### 2.6. Data Acquisition

The mechanical properties were tested in accordance with the Standard for Test Methods of Mechanical Properties of Ordinary Concrete (GB50081-2002) [32].

#### 2.6.1. Cubic Compressive Strength

Equation (1) was used as the cubic compressive strength formula:

(1) $$f_{cu}=\frac{F}{A}$$

where *f_cu_* is the cube compressive strength of the specimen (MPa); *F* is the destructive load of the specimen (N); and *A* is the area of the specimen subjected to force (mm^2^).

#### 2.6.2. Splitting Tensile Strength

The adopted splitting tensile strength formula is shown in Equation (2):

(2) $$f_{ts}=0.637\frac{F}{A}$$

where *f_ts_* is the cubic compressive strength of the specimen (MPa); *F* is the destructive load of the specimen (N); and *A* is the area of the specimen subjected to force (mm^2^).

#### 2.6.3. Flexural Strength

The formula used for flexural strength is outlined in Equation (3):

(3) $$f_{f}=\frac{Fl}{bh^{2}}$$

where *f_f_* is the specimen cubic compressive strength (MPa); *F* is the specimen’s destructive load (N); *l* is the span between supports (mm); *h* is the specimen cross-section height (mm); and *b* is the specimen cross-section width (mm).

#### 2.6.4. Impact Strength

The impact strength was calculated using Equations (5) and (6):

(4) $$w_{i}=N_{i}\cdot mg\cdot h$$

(5) $$\mu =(N_{2}-N_{1})/N_{1}$$

where *w_i_* is the impact dissipation energy of the specimen (J); *N_i_* is the number of free falls of the steel ball; *m* is the mass of the steel ball (kg); *g* is the acceleration of gravity (9.8 m/s^2^); *h* is the height of the free fall of the steel ball (mm); and *μ* is the ductility ratio.

## 3. Results and Analysis

Table 8 shows the cubic compressive strength, splitting tensile strength, and flexural strength of the three benchmark concrete groups with different water–cement ratios and the nine blended straw–scrap-steel-fiber concrete groups. It can be seen that the cubic compressive strength, splitting tensile strength, and flexural strength of the nine groups of blended straw–waste-steel-fiber concrete were greater than the strength of the benchmark group of concrete with the same water–cement ratio, and all of them satisfied the strength of C40 concrete. Among them, the cubic compressive strength of group 5 was the highest, which was 14.96% higher than that of the benchmark concrete with the same water–cement ratio. The splitting tensile strength of group 5 was also the highest, which was 42.90% higher than that of the benchmark concrete with the same water–cement ratio. Meanwhile, the flexural strength of group 2 was the highest, which was 16.30% higher than that of the benchmark concrete with the same water–cement ratio.

### 3.1. Cube Compressive Strength Test

#### 3.1.1. Cubic Compressive Strength Damage Patterns

**Figure 5:**
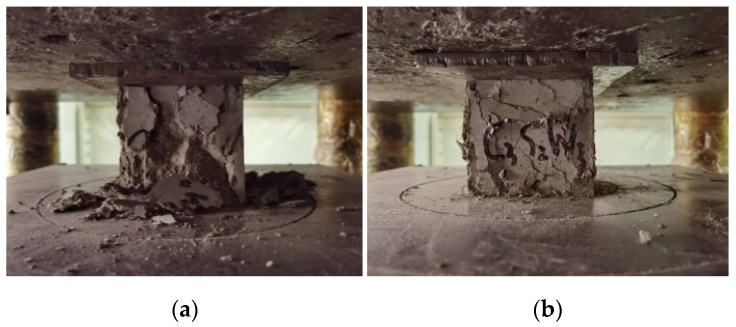
Damage patterns observed in the cubic compressive strength test. (**a**) Plain concrete group; (**b**) fiber concrete group.

Figure 5 shows the final damage patterns after the cubic compressive test of the plain concrete and blended straw–waste-steel-fiber concrete. The damage process of the concrete showed brittle damage with cracks and surface spalling in the specimens, and a shorter damage process was experienced when ultimate load-capacity damage occurred. For the specimen of the fiber concrete group in the early loading period, the cracks started to appear at the upper and lower edges and gradually enlarged; in the middle of the loading period, the specimen began to expand around the perimeter and the surface spalled, while at the end of the loading period, the cracks enlarged and penetrated through each other until destruction. The main reason for this is that the corn straw fibers and scrap steel fibers connected to the concrete at the cracks, and their connecting force made the fiber concrete better than the plain concrete as a whole. The scrap steel fibers, because of the shape of the curved concrete, formed a certain anchoring effect that allowed the concrete to resist deformation after it was subjected to pressure and underwent destruction. The corn straw fibers, being softer and having undergone surface modification to increase their fluffiness, could fill the small pores in the concrete, preventing the expansion of cracks [34].

#### 3.1.2. Cubic Compressive Strength Analysis

As can be seen in Table 9, the degree of each factor’s influence on the compressive strength of the concrete was obtained using polar analysis, as follows: scrap steel fibers (3.50) > water–cement ratio (3.07) > corn straw fibers (1.96). From the analysis of variance of the compressive strength shown in Table 10, it is clear that all three factors had a significant influence on the compressive strength.

**Figure 6:**
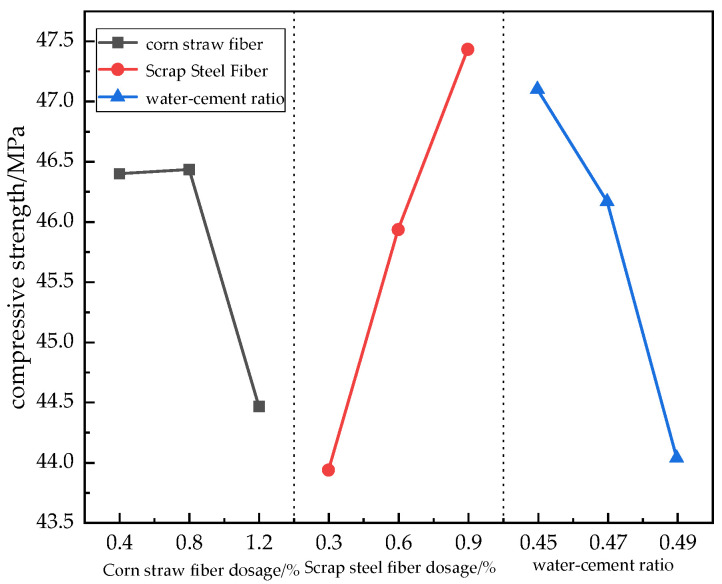
Effects of different factors on the compressive strength.

As can be seen in Figure 6, when the volume dosage of scrap steel fibers was from 0.3% to 0.6%, the compressive strength increased by 4.55%, and the steel fiber volume dosages of 0.6% to 0.9% increased the compressive strength by 3.27%. When the water–cement ratio was from 0.45 to 0.47, the compressive strength decreased by 1.97%, and this decrease in compressive strength was 4.64% when the water–cement ratio was from 0.47 to 0.49. The corn straw fiber dosages of 0.4% to 0.8% increased the compressive strength by 0.06%, and the corn straw fiber volume dosages of 0.8% to 1.2% decreased the compressive strength by 4.22%. The main factor affecting the compressive strength was the dosage of scrap steel fibers, and it can be seen from Figure 6 that the compressive strength was highest when this dosage was 0.9%. This is because scrap steel fibers play the role of a load-bearing skeleton in the concrete to make the internal structure more compact, and their good mechanical properties help the concrete bear some of the pressure. When the dosage of corn straw fibers was 0.8%, the compressive strength of the concrete slightly improved, and corn straw fibers 2–3 cm in length were able to improve the compressive strength because they could prevent the production of small cracks. However, corn straw fiber doping at more than 0.8% decreased the compressive strength, because too much corn straw fiber doping reduces the amount of cement per unit volume, resulting in a lower strength of the specimen. At the same time, too much corn straw fiber doping causes agglomeration, affecting the dispersion of fibers and their combination with the cement. Having too many fibers in the slurry produced too many small holes, so the pore wall became thin, while the density also reduced, affecting its compressive strength. The concrete with the lowest water–cement ratio had the highest strength, indicating that the effect of straw water absorption on the water–cement ratio was not significant for this mixing range, and the compressive strength decreased with increases in the water–cement ratio. In conclusion, the optimal mixing parameters were a 0.8% volume of corn straw fibers, a 0.9% volume of scrap steel fibers, and a water–cement ratio of 0.45, with which the inclusion of both fibers increased the compressive strength of the cube, showing a positive mixing effect.

### 3.2. Splitting Tensile Strength Test

#### 3.2.1. Splitting Tensile Strength Damage Patterns

**Figure 7:**
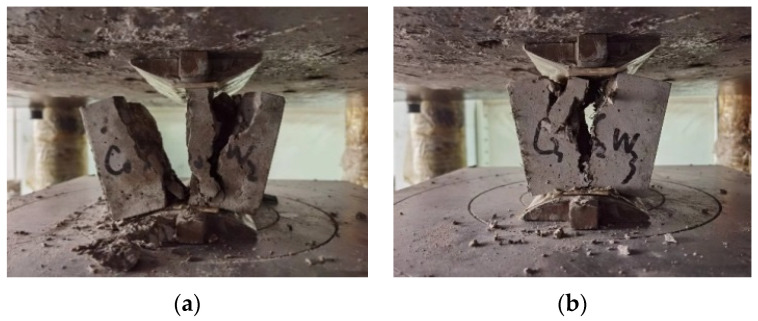
Splitting tensile test damage patterns. (**a**) Plain concrete group; (**b**) fiber concrete group.

Figure 7 shows the damage pattern in the splitting tensile tests of plain concrete and blended straw–scrap-steel-fiber concrete. The plain concrete underwent sudden brittle damage with the increasing load, characterized by the appearance of a large crack through the cross-section, thus completely splitting the concrete. The split cross-section showed that some fragments of this concrete had been stripped out, as shown in Figure 7a. In contrast, the splitting damage of the fiber concrete group mainly produced two cracks of the Y or H type, which made it difficult for the specimen to completely split apart and allowed it to maintain better integrity, with obvious damage precursors, as shown in Figure 7b. The fibers improved the splitting and tensile properties of the concrete, while the connecting force generated by the fibers and the concrete prevented cracks from occurring. Many fibers can still be seen connected at the cracks, and the larger the fiber portion in the admixture, the smaller the cracks in the specimens. The scrap steel fibers showed superior mechanical properties due to their bent shape, forming an anchoring effect in the concrete, absorbing part of the tensile force and preventing crack expansion. The corn straw fibers connected the concrete, preventing the separation of parts during secondary damage, resulting in a tighter connection between the materials.

#### 3.2.2. Splitting Tensile Strength Analysis

**Figure 8:**
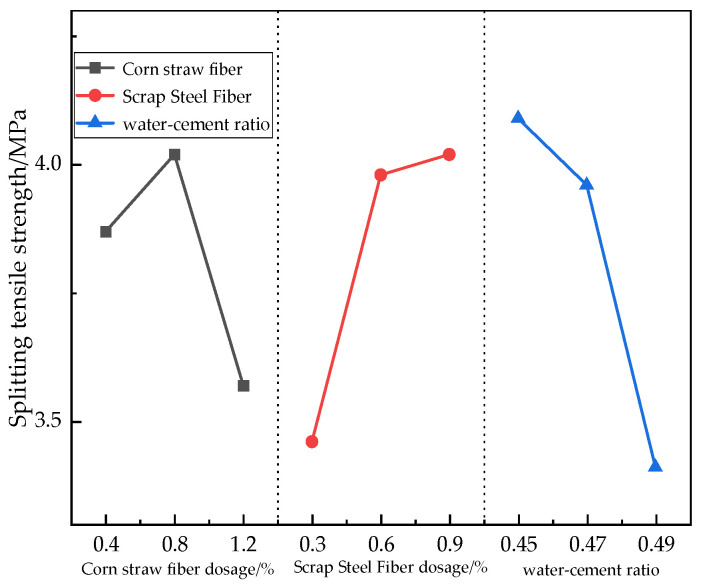
Effects of different factors on the splitting tensile strength.

The degree of influence of each factor on the splitting tensile strength of concrete was obtained via polar analysis, as follows (Table 11): water–cement ratio (0.68) > scrap steel fibers (0.56) > corn straw fibers (0.45). From the analysis of variance in Table 12, it can be seen that the size of the water–cement ratio and the dosage of scrap steel fibers have a significant effect on the splitting tensile strength of concrete, while the dosage of corn straw fibers does not. As can be seen in Figure 8, the splitting tensile strength decreased by 3.18% when the water–cement ratio went from 0.45 to 0.47 and by 13.89% when it went from 0.47 to 0.49. The splitting tensile strength increased by 3.88% when the corn straw fiber volume dosage was from 0.4% to 0.8% and decreased by 11.19% when this volume dosage was from 0.8% to 1.2%. The splitting tensile strength increased by 15.03% when the scrap steel fiber volume dosage was from 0.3% to 0.6% and by 1.01% when this volume dosage was from 0.6% to 0.9%. Changes in the water–cement ratio had a significant effect on the splitting tensile strength; with a small water–cement ratio, a larger concentration of cement paste was generated, the adhesive area of the colloid and aggregate became large, and the splitting tensile strength of concrete increased. The scrap steel fibers still provided good enhancement of the mechanical effects. When the dosage of waste steel fibers exceeded 0.6%, the slope of the tensile strength curve decreased, indicating that further increasing the dosage of scrap steel fibers does not significantly improve the splitting tensile strength. The optimal amount of corn straw fibers in concrete mixing results in a more uniform dispersal, creating a three-dimensional chaotic distribution internally. This improves the generation of cracks and the formation of friction and mechanical occlusion within the concrete so as to effectively transfer the load. In the tensile process, if the bonding force is greater than the tensile strength of the fibers themselves, the fibers first break and destroy, and if the bonding force is less than the tensile strength, bonding damage occurs. The optimal dosage of corn straw fibers was 0.8% and that of scrap steel fibers was 0.9%, while the optimal water–cement ratio was 0.45. Under these dosages, the hybrid fiber addition prevented the development of small cracks inside the concrete and relieved the stress concentration phenomenon at the crack tip in the concrete so as to increase its splitting tensile strength.

### 3.3. Flexural Strength Test

#### 3.3.1. Flexural Strength Damage Pattern

**Figure 9:**
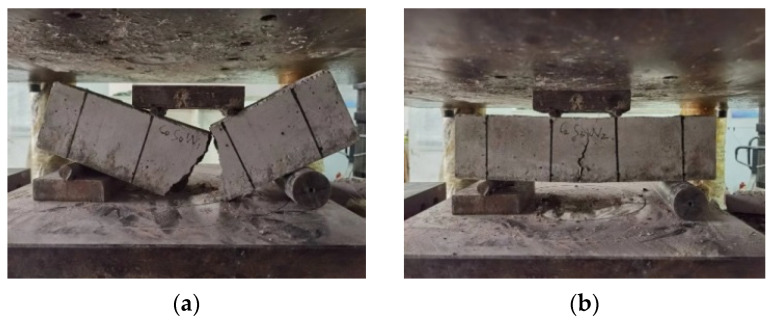
Fracture strength test damage patterns. (**a**) Plain concrete group; (**b**) fiber concrete group.

Figure 9 shows the damage patterns of the plain concrete and blended straw–waste-steel-fiber concrete specimens observed in the flexural test. In the plain concrete, in the process of force fracturing, cracks appeared after the specimen rapidly fractured into two parts, and obvious crushing sounds could be heard, which manifested as brittle damage. The concrete mixed with the fiber group initially developed small cracks around the center of the bottom of the cross-section under the tested force. With the gradual increase in load, the cracks slowly extended upward and expanded until reaching the peak load. The specimen still maintained its overall structure after being damaged, showing ductile damage characteristics. The two kinds of fibers within the concrete withstood a portion of the bending tensile force, enhancing the concrete’s ability to withstand ultimate breaking stress. Observing the cracks, it was found that the waste steel fibers experienced pull-out damage rather than pull-off damage. During this destructive process of fiber pull-out, a significant amount of stress is absorbed, delaying the bending and pull-out destruction of the specimen. This fiber mixing made the concrete more compact, thereby preventing rapid crack propagation during specimen bending.

#### 3.3.2. Flexural Strength Analysis

**Figure 10:**
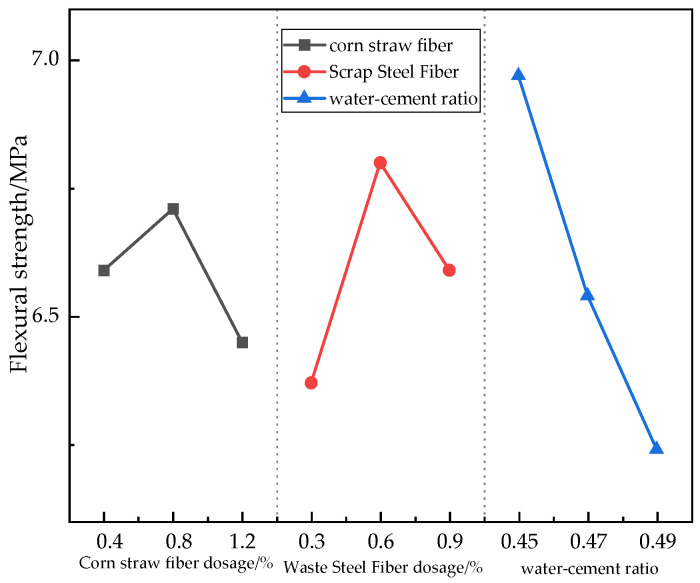
Effects of different factors on the flexural strength.

From the polar analysis (Table 13), the degree of each factor’s influence on the flexural strength of concrete was obtained as follows: water–cement ratio (0.73) > scrap steel fibers (0.43) > corn straw fibers (0.26). From the analysis of variance (ANOVA) in Table 14, it can be seen that the magnitude of the water–cement ratio and the admixture of scrap steel fibers had a significant effect on the flexural strength of the concrete, while the admixture of corn straw fibers did not. From Figure 10, it can be seen that the flexural strength decreased by 6.17% when the water–cement ratio increased from 0.45 to 0.47 and by 4.59% when it increased from 0.47 to 0.49. A scrap steel fiber volume dosage of 0.3% to 0.6% increased the flexural strength by 6.75%, while dosages from 0.6% to 0.9% decreased the flexural strength by 3.09%. A corn straw fiber volume of 0.4% to 0.8% increased the flexural strength by 1.82%, while a corn straw fiber volume of 0.8% to 1.2% decreased the flexural strength by 3.87%. Changes in the water–cement ratio had a significant effect on the flexural strength of the concrete—the higher the water–cement ratio, the lower the flexural strength of the concrete. The reason for this is that as the water–cement ratio increased, it led to more internal pores, and the porosity of the concrete increased, which decreases its flexural strength. In the concrete mixed with an appropriate amount of waste material, the steel fibers provided sufficient holding force. In the specimen subjected to external forces that resulted in cracks, pulling out the scrap steel fibers required a lot of energy, which allowed it to effectively resist damage [35,36]. When the dosage of scrap steel fibers exceeded 0.6%, the flexural strength decreased. The reason for this is that continuing to increase the dosage of scrap steel fibers led to a certain amount of balling, affecting its mechanical enhancement effect. The admixture of corn straw fibers played a bridging role in the concrete at a dosage of up to 0.8%, enhancing the ductility of the concrete, improving its flexural properties, improving the toughness of the concrete, and inhibiting the emergence and development of fine cracks. However, the enhancement of its flexural strength was not obvious because of its inherently low modulus of elasticity. When too many straw fibers were mixed in, they formed excessive “bridges”, reducing the hydration reaction products and thereby decreasing the flexural strength. For flexural properties, the optimum dosage of corn straw fibers was 0.8%, the optimum dosage of scrap steel fibers was 0.6%, and the optimum water–cement ratio was 0.45.

### 3.4. Impact Strength Test

As can be seen from the data in Table 15, the second group had the highest rates of impact energy dissipation upon initial and final cracking, which were 212.90% and 228.03% higher than that of the plain concrete group with the same water–cement ratio, respectively. At the same time, the impact energy observed at the initial and final cracking of the fiber-added group was greater than that observed for the plain concrete group with the same water–cement ratio. This indicates that the incorporation of corn straw fibers and scrap steel fibers helps to improve the impact resistance of concrete. The ductility ratios of the fiber group were also greater than those of the plain concrete group, and the incorporation of the fibers improved the impact toughness of the concrete [37].

#### 3.4.1. Impact Strength Damage Pattern

**Figure 11:**
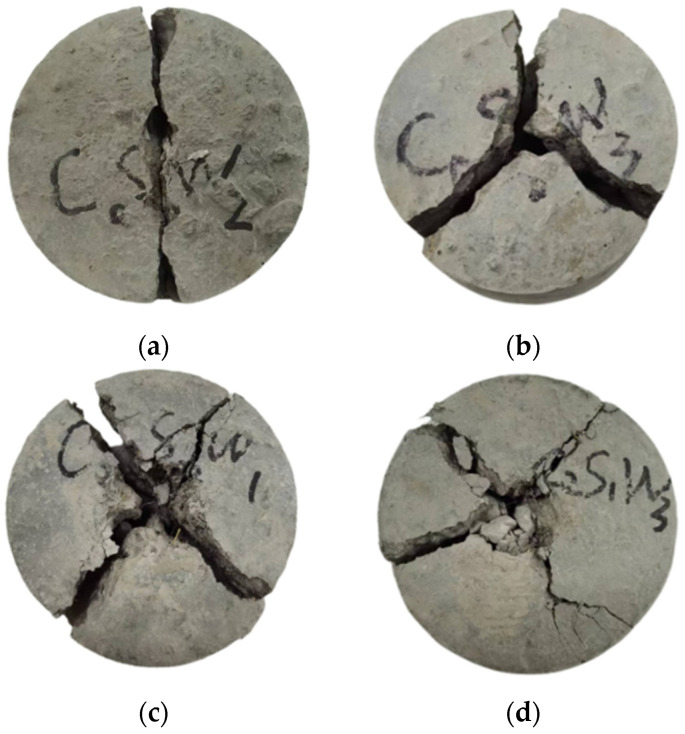
Specimen impact damage patterns. (**a**) Through failure; (**b**) cracking in three parts; (**c**) cracking in four cracks; (**d**) cracking in four parts.

From Figure 11a,b, it can be seen that the impact damage of the plain concrete specimen mostly produced one or three cracks. When there was penetrative damage, the specimen cracked into two uniform parts, and when there were three cracks, it cracked more uniformly into three parts. As can be seen from Figure 11c,d, the incorporation of fibers increased the number of impacts sustained by the specimens compared to the benchmark group, resulting in mostly four final cracks. The incorporation of scrap steel fibers and corn straw fibers caused plastic damage to the concrete, producing smaller cracks. The area of the impact marks in the center of the fiber group was also more pronounced than that of those in the baseline group of specimens since the concrete in the fiber group required multiple impacts before it was damaged. The cracks observed at the connection of the scrap steel fibers and corn straw fibers were mainly controlled by the scrap steel fibers, preventing continued expansion of the cracks [38]. The corn straw fibers themselves were softer but also had the ability to absorb the impact energy, which further increased the number of times specimen impact damage occurred.

#### 3.4.2. Impact Strength Analysis

From the polar analysis (Table 16), the degree of each factor’s influence on the impact strength of concrete was as follows: scrap steel fibers (2465.5) > corn straw fibers (1679.5) > water–cement ratio (1518.2). From the analysis of variance (ANOVA) in Table 17, it is clear that the admixture of both fibers had a significant effect on the impact strength of the concrete.

**Figure 12:**
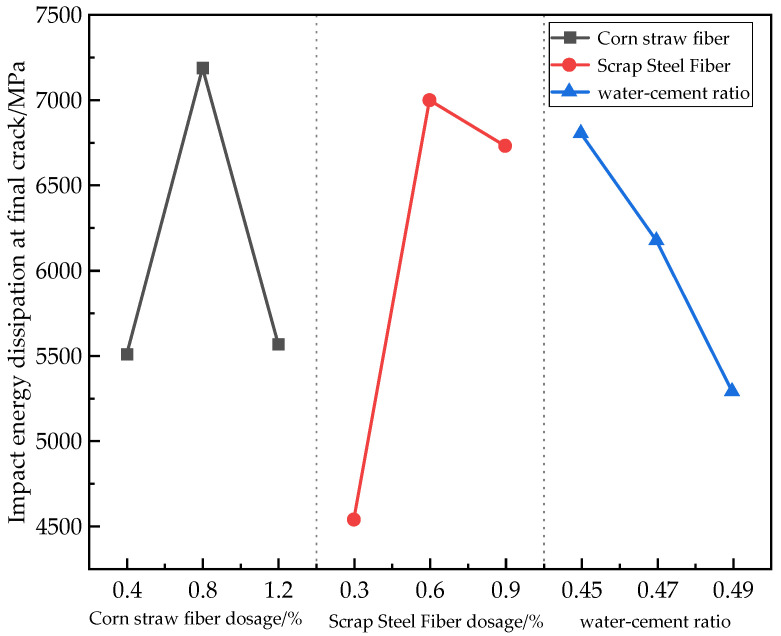
Effects of different factors on the impact strength.

It can also be seen from Figure 12 that there was a 54.37% increase in the impact energy consumption for scrap steel fiber volume dosages from 0.3% to 0.6% and a 3.84% decrease for those volume dosages from 0.6% to 0.9%. The impact energy consumption increased by 30.49% when the volume dosage of corn straw fibers went from 0.4% to 0.8% and decreased by 22.52% when it went from 0.8% to 1.2%. The impact energy consumption was reduced by 9.28% when the water–cement ratio went from 0.45 to 0.47 and by 14.36% when it went from 0.47 to 0.49. The scrap steel fibers resisted crack enlargement due to impact, and when subjected to external forces for continuous impact, the impact energy was transferred to the corn straw fibers and thus preferentially absorbed and dispersed. The straw fibers had a good deformation capacity compared to the coarse and fine aggregates in the concrete, resulting in a large damping coefficient, and the impact energy caused by its power loading was good [39]. Meanwhile, the surface of the corn straw fibers after NaOH treatment was rough and distributed with fine filamentous tissues, and this further improved the specimen’s ability to absorb the impact energy. For the impact strength, the optimum dosage of corn straw fibers was 0.8%, the optimum dosage of scrap steel fibers was 0.6%, and the optimum water–cement ratio was 0.45.

## 4. Microanalysis

**Figure 13:**
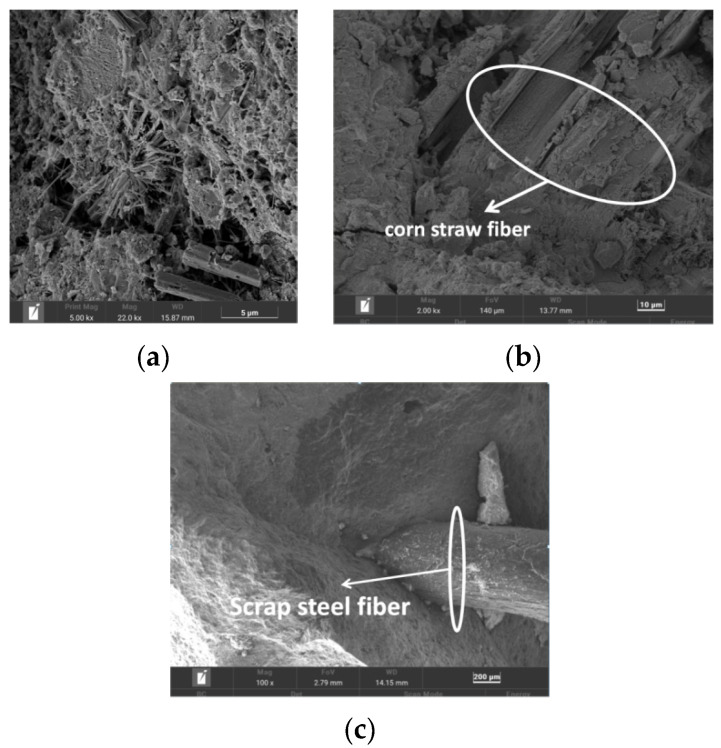
Microanalysis. (**a**) C_0_S_0_W_1_; (**b**) corn straw fibers; (**c**) scrap steel fibers.

C_0_S_0_W_1_ and C_2_S_3_W_2_ were sampled separately for microanalysis. As observed in Figure 13a, the hydration products of the standard maintenance of 28-day C_0_S_0_W_1_ were mainly reticulated flocculent C-S-H gels, needle- and rod-shaped ettringite (AFt), and hexagonal prismatic CH crystals. Most of this needle- and rod-shaped AFt was distributed in the tiny cracks of the concrete, and AFt can very easily generate expansion pressure inside a specimen [40,41,42], so the mechanical properties of the unmixed fiber series were lower than those of the mixed fiber series. As can be seen from Figure 13b,c, the corn straw fibers and scrap steel fibers formed a better bond with the cement slurry, which formed an integral part of the slurry, effectively filled the pores of the specimen, and enhanced the integrity of the specimen, resulting in better mechanical properties [43,44,45].

## 5. Hierarchical Analysis of the Mechanical Parts of the Blended Straw–Waste-Steel-Fiber Concrete

### 5.1. Significance of Hierarchical Analysis

**Figure 14:**
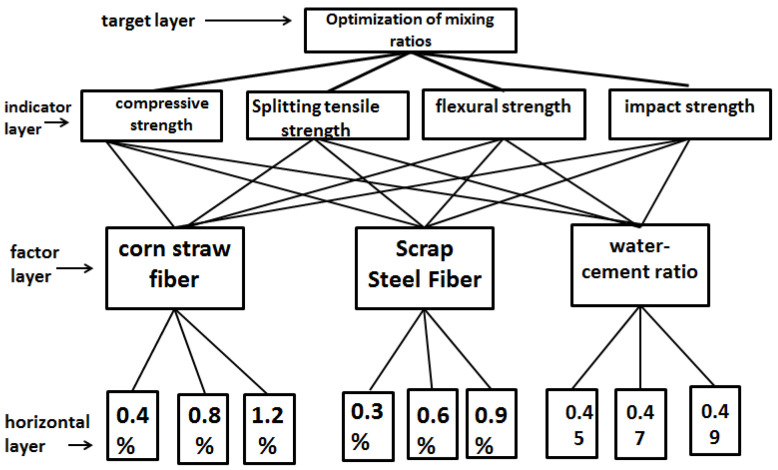
Diagram of hierarchical analysis structure.

The optimum level and significance of each factor for the compressive strength, splitting tensile strength, flexural strength, and impact resistance can be obtained through polar analysis and ANOVA. However, the optimum level of each factor under different mechanical properties varies, and there are some limitations in using polar analysis and ANOVA alone, so in order to optimize the optimum mix ratio, hierarchical analysis was used to reach the objective, as shown in Figure 14.

### 5.2. Model Building

(1) Establishment of a matrix of impact effects at the indicator level:

The average value of factor *j* at the *i*th level is *k_ij_*. According to the principle that a higher calculation result indicates better performance, we let *K_ij_* = *k_ij_*, and establish the matrix shown in Equation (6).

(6) $$M=\left(\begin{array}{ccc} \begin{array}{l} K_{1A}\\ K_{2A}\\ K_{3A} \end{array} & & \\ & \begin{array}{l} K_{1B}\\ K_{2B}\\ K_{3B} \end{array} & \\ & & \begin{array}{l} K_{1C}\\ K_{2C}\\ K_{3C} \end{array} \end{array}\right)$$

(2) Creating an impact effect matrix for the factor stratum:

Let $t_{j}=1/\sum_{i=1}^{3}K_{ij}\left(i=1,2,3;j=A,B,C\right)$. The matrix of influence effects for the factor layers is thus obtained as shown in Equation (7).

(7) $$T=\left(\begin{array}{ccc} t_{A} & & \\ & t_{B} & \\ & & t_{C} \end{array}\right)$$

(3) Establishment of a matrix of impact effects at the horizontal level:

Let $c_{i}=R_{i}/\sum_{i=1}^{3}R_{i}\left(i=1,2,3\right)$. The impact effect matrix for the horizontal layer is then obtained as shown in Equation (8).

(8) $$\Omega =MTC^{T}$$

(9) $$\bar{\Omega }=\frac{1}{6}\sum_{l=1}^{6}\Omega_{l}$$

**Figure 15:**
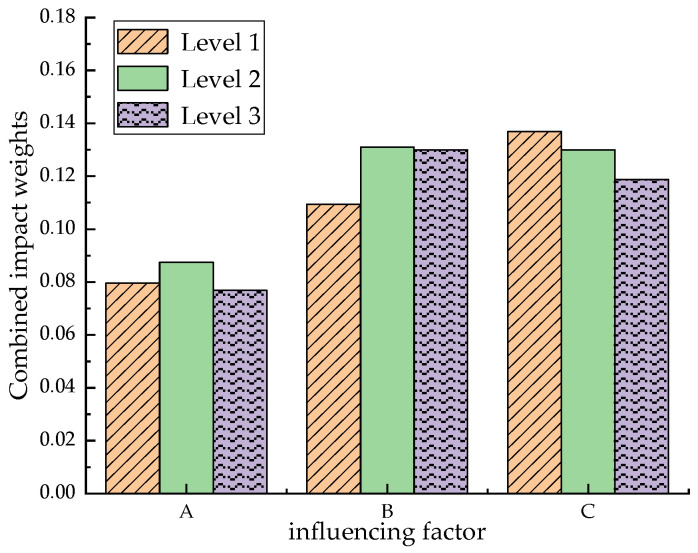
Hierarchical structure diagram of the blended straw–waste-steel-fiber concrete AHP model.

The orthogonal test data for the mechanical part were calculated according to the above formulas to obtain the influence weights of each factor and the combined influence weights, which are shown in Table 18. The maximum value in the combined influence weights derived from the different factors represents the optimum level of that factor in the blended straw–scrap-steel-fiber concrete group. In order to be able to compare the combined influence weights of the factors more intuitively, a histogram was plotted, as shown in Figure 15, and the optimal fit ratio was derived from a comparison of the results.

### 5.3. Optimal Mixing Ratio

Figure 15 shows that the second level of the combined influence of factor A had the highest weight, the second level of the combined influence of factor B had the highest weight, and the first level of the combined influence of factor C had the highest weight. Based on the weights, the optimal combination comprised 0.8% by volume of admixture of corn straw fibers and 0.6% by volume of admixture of scrap steel fibers, with a water–cement ratio of 0.45.

## 6. Conclusions

In this study, certain aspects of concrete mixtures, such as compressive strength and impact strength, were investigated. The compressive strength of concrete made with corn stover fibers and waste steel fibers achieved the highest value of 48.4 MPa; its splitting tensile strength achieved the highest value of 4.43 MPa, and its flexural strength achieved the highest value of 7.35 MPa. Upon its initial cracking, the impact strength of this concrete made with corn stover fibers and waste steel fiber concrete achieved the highest value of 7819.6 J, and at the final cracking, its impact energy achieved the highest value of 8726.6 J.

(1) The compressive strength, splitting tensile strength, flexural strength, and impact resistance of the blended straw–scrap-steel-fiber concrete were stronger than those of the benchmark concrete with the same water–cement ratio. Combined with the microscopic images, it can be seen that the corn straw fibers and waste steel fibers effectively filled the pores and improved the densification of the matrix, which indicates that the hybrid fibers had a positive mixing benefit on the mechanical properties of the concrete.

(2) Based on the analysis of variance, the corn straw fiber admixture was a significant influencing factor for the cubic compressive strength and impact resistance of the concrete. The scrap steel fiber admixture was a significant influencing factor for the compressive strength, splitting tensile strength, flexural strength, and impact resistance of the concrete. Additionally, the water–cement ratio was a significant influencing factor for the compressive strength, splitting tensile strength, and flexural strength of the concrete.

(3) Based on the analysis of extreme differences, in terms of cubic compressive strength, the optimum dosage of corn straw fibers was 0.8%, the optimum dosage of scrap steel fibers was 0.9%, and the optimum water–cement ratio was 0.45, which improved the cubic compressive strength by 14.96% compared with the basic group. In terms of the splitting tensile strength, the optimum dosage of corn straw fibers was 0.8%, the optimum dosage of scrap steel fibers was 0.9%, and the optimum water–cement ratio was 0.45, which led to the splitting tensile strength being 42.90% higher than that observed in the basic group. In terms of flexural strength, the optimum dosage of corn straw fibers was 0.8%, the optimum dosage of scrap steel fibers was 0.6%, and the optimum water–cement ratio was 0.45, which improved the flexural strength by 16.30% compared to the basic group. In terms of impact resistance performance, the optimum dosage of corn straw fibers was 0.8%, the optimum dosage of scrap steel fibers was 0.6%, and the optimum water–cement ratio was 0.45; with these values, the impact energy consumption of final cracking was improved by 228.03% compared to the basic group.

(4) By establishing a model using the hierarchical analysis method and synthesizing the influence weights of each level of each factor under different mechanical properties, the optimal mixing ratio was derived as being 0.8% corn straw fiber mixing and 0.6% waste steel fiber mixing, with a water–cement ratio of 0.45. Further analyses of these optimal blending amounts could provide some insights into the promotion of corn straw fibers and scrap steel fibers.

## Figures and Tables

**Table 1:** Physical indicators of the sand.

Fineness Modulus	Apparent Density (kg/m^3^)	Packing Density (kg/m^3^)	Mud Content (%)
2.41	2640	1868	2

**Table 2:** Physical indexes of the natural aggregate.

Particle Gradation (mm)	Mud Content (%)	Indicator of Crushing (%)	Apparent Density (kg/m^3^)	Packing Density (kg/m^3^)
5–20	0.3	9.4	2573	1460

**Table 3:** Chemical composition and content of the cement.

Chemical Composition	SiO_2_	Al_2_O_3_	Fe_2_O_3_	CaO	SO_3_	MgO
Quantity contained (%)	19.6	6.5	1.03	66.3	2.5	0.7

**Table 4:** Partial performance parameters of the corn straw fibers.

Hemicellulose/%	Cellulose/%	Lignin/%	Crude Protein/%	Length/mm	Density/kg/m^3^	Water Absorption/%	pH
19.17	47.72	9.47	23.64	20~30	290	5.5	7.1

**Table 5:** Performance parameters of the scrap steel fibers.

Caliber/mm	Length/mm	L/D Ratio	Density/kg/m^3^	Tensile Strength/MPa	Modulus of Elasticity/MPa
0.5~0.6	10~30	65	7800	1345	220

**Table 6:** Concrete mixtures.

Group	Water/kg/m^3^	Cement/kg/m^3^	Fine Aggregate/kg/m^3^	Coarse Aggregate/kg/m^3^	Straw Content/%	Scrap Steel Fiber Content/%	Water-Reducing Agent/kg/m^3^	Water–Cement Ratio
C_1_S_1_W_1_	195	433	620	1152	0.4	0.3	2.17	0.45
C_2_S_2_W_1_	195	433	620	1152	0.8	0.6	2.17	0.45
C_3_S_3_W_1_	195	433	620	1152	1.2	0.9	2.17	0.45
C_1_S_2_W_2_	195	415	626	1164	0.4	0.6	2.08	0.47
C_2_S_3_W_2_	195	415	626	1164	0.8	0.9	2.08	0.47
C_3_S_1_W_2_	195	415	626	1164	1.2	0.3	2.08	0.47
C_1_S_3_W_3_	195	398	632	1175	0.4	0.9	1.99	0.49
C_2_S_1_W_3_	195	398	632	1175	0.8	0.3	1.99	0.49
C_3_S_2_W_3_	195	398	632	1175	1.2	0.6	1.99	0.49
C_0_S_0_W_1_	195	433	620	1152	0	0	2.17	0.45
C_0_S_0_W_2_	195	415	626	1164	0	0	2.08	0.47
C_0_S_0_W_3_	195	398	632	1175	0	0	1.99	0.49

**Table 7:** Orthogonal experiment table.

Level	Corn Straw Fiber Dosage (Factor A)/%	Scrap Steel Fiber Dosage (Factor B)/%	Water–Cement Ratio (Factor C)
1	0.4	0.3%	0.45
2	0.8	0.6%	0.47
3	1.2	0.9%	0.49

**Table 8:** Mechanical test results of the blended straw–waste-steel-fiber concrete.

Group	Serial Number	A	B	C	Cubic Compressive Strength/MPa	Splitting Tensile Strength/MPa	Flexural Strength/MPa
		Corn Straw Fiber Dosage/%	Scrap Steel Fiber Dosage/%	Water–Cement Ratio			
1	C_1_S_1_W_1_	1 (0.4)	1 (0.3)	1 (0.45)	45.8	3.85	6.73
2	C_2_S_2_W_1_	2 (0.8)	2 (0.6)	1 (0.45)	47.8	4.42	7.35
3	C_3_S_3_W_1_	3 (1.2)	3 (0.9)	1 (0.45)	47.7	4.01	6.84
4	C_1_S_2_W_2_	1 (0.4)	2 (0.6)	2 (0.47)	47.2	4.13	6.75
5	C_2_S_3_W_2_	2 (0.8)	3 (0.9)	2 (0.47)	48.4	4.43	6.64
6	C_3_S_1_W_2_	3 (1.2)	1 (0.3)	2 (0.47)	42.9	3.31	6.22
7	C_1_S_3_W_3_	1 (0.4)	3 (0.9)	3 (0.49)	46.2	3.62	6.28
8	C_2_S_1_W_3_	2 (0.8)	1 (0.3)	3 (0.49)	43.1	3.22	6.15
9	C_3_S_2_W_3_	3 (1.2)	2 (0.6)	3 (0.49)	42.8	3.4	6.3
10	C_0_S_0_W_1_	0	0	1 (0.45)	45.5	3.43	6.32
11	C_0_S_0_W_2_	0	0	2 (0.47)	42.1	3.10	6.11
12	C_0_S_0_W_3_	0	0	3 (0.49)	40.1	3.06	6.03

**Table 9:** Compressive strength range analysis results.

	Compressive Strength/MPa			
Factor	K1	K2	K3	R
A	46.40	46.43	44.47	1.96
B	43.93	45.93	47.43	3.50
C	47.10	46.17	44.03	3.07

**Table 10:** Compressive strength variance analysis results.

Norm	Factor	Degrees of Freedom (DF)	Sum of Squared Deviations (SS)	Mean Square (MS)	Statistical Value (F)	Probability of Significance (P)	Salience Status
Compressive strength	A	2	7.607	3.803	30.838	0.0314	Salient
	B	2	18.500	9.250	75.000	0.0134	Salient
	C	2	14.827	7.413	60.108	0.0164	Salient
	e	2	0.247	0.123			
	T	8	41.180	5.148			

**Table 11:** Splitting tensile strength range analysis results.

	Splitting Tensile Strength/MPa			
Factor	K1	K2	K3	R
A	3.87	4.02	3.57	0.45
B	3.46	3.98	4.02	0.56
C	4.09	3.96	3.41	0.68

**Table 12:** Splitting tensile strength variance analysis results.

Norm	Factor	Degrees of Freedom (DF)	Sum of Squared Deviations (SS)	Mean Square (MS)	Statistical Value (F)	Probability of Significance (P)	Salience Status
Splitting tensile strength	A	2	0.313	0.157	13.329	0.070	Non-salient
	B	2	0.589	0.294	25.068	0.038	Salient
	C	2	0.776	0.388	33.049	0.029	Salient
	e	2	0.023	0.012			
	T	8	1.702	0.213			

**Table 13:** Range analysis of the flexural strength of the blended straw–waste-steel-fiber concrete.

	Flexural Strength/MPa			
Factor	K1	K2	K3	R
A	6.59	6.71	6.45	0.26
B	6.37	6.80	6.59	0.43
C	6.97	6.54	6.24	0.73

**Table 14:** Variance analysis results for the bending strength of the blended straw–waste-steel-fiber concrete.

Norm	Factor	Degrees of Freedom (DF)	Sum of Squared Deviations (SS)	Mean Square (MS)	Statistical Value (F)	Probability of Significance (P)	Salience Status
Flexural strength	A	2	0.101	0.051	18.478	0.051	Non-salient
	B	2	0.282	0.141	51.320	0.019	Salient
	C	2	0.810	0.405	147.502	0.007	Salient
	e	2	0.005	0.003			
	T	8	1.198	0.150			

**Table 15:** Impact test results for the blended straw–waste-steel-fiber concrete.

Specimen Number	A	B	C	Impact Resistance Test Results				
	Corn Straw Fiber Content/%	Scrap Steel Fiber Content/%	Water– Cement Ratio	Average Number of Impacts/Times (Initial Cracking)	Impact Energy Consumption/J (Initial Cracking)	Average Number of Impacts/Times (Final Cracking)	Impact Energy Dissipation/J (Final Cracking)	Ductility Ratio/%
C_1_S_1_W_1_	0.4	0.3	0.45	205	4131.5	224	4514.4	9.27
C_2_S_2_W_1_	0.8	0.6	0.45	388	7819.6	433	8726.6	11.60
C_3_S_3_W_1_	1.2	0.9	0.45	318	6408.9	356	7174.7	11.95
C_1_S_2_W_2_	0.4	0.6	0.47	299	6026.0	335	6751.5	12.04
C_2_S_3_W_2_	0.8	0.9	0.47	345	6953.0	385	7759.2	11.59
C_3_S_1_W_2_	1.2	0.3	0.47	184	3708.3	199	4010.6	8.15
C_1_S_3_W_3_	0.4	0.9	0.49	241	4857.0	261	5260.1	8.30
C_2_S_1_W_3_	0.8	0.3	0.49	238	4796.6	252	5078.7	5.88
C_3_S_2_W_3_	1.2	0.6	0.49	253	5098.9	274	5522.1	8.30
C_0_S_0_W_1_	0	0	0.45	124	2499.1	132	2660.3	6.45
C_0_S_0_W_2_	0	0	0.47	105	2116.1	109	2196.8	3.81
C_0_S_0_W_3_	0	0	0.49	85	1713.1	88	1773.5	3.53

**Table 16:** Impact energy range analysis of the final cracks in the impact tests.

Impact Energy Consumption/J				
Factor	K1	K2	K3	R
A	5508.7	7188.2	5569.1	1679.5
B	4534.6	7000.1	6731.3	2465.5
C	6805.2	6173.8	5287.0	1518.2

**Table 17:** Impact energy dissipation analysis results for the impact testing.

Norm	Factor	Degrees of Freedom (DF)	Sum of Squared Deviations (SS)	Mean Square (MS)	Statistical Value (F)	Probability of Significance (P)	Salience Status
Impact energy consumption/J	A	2	5,445,497.829	2,722,748.914	19.462	0.049	Salient
	B	2	10,976,472.22	5,488,236.109	39.229	0.025	Salient
	C	2	3,490,187.632	1,745,093.816	12.474	0.074	Non-salient
	e	2	279,807.119	139,903.559			
	T	8	20,191,964.8	2,523,995.6			

**Table 18:** Influence weights of the mechanical properties of various factors and combined influence weights.

Factor	Level	Stress Tolerance Weighting	Splitting Tensile Weighting	Flexural Weighting	Impact Energy Dissipation Weights for Final Cracking	Combined Impact Weights
A (corn straw fibers)	1	0.0779	0.0898	0.0609	0.0894	0.0795
	2	0.0779	0.0935	0.0621	0.1167	0.0875
	3	0.0746	0.0830	0.0597	0.0904	0.0769
B (scrap steel fibers)	1	0.1312	0.1000	0.0981	0.1081	0.1094
	2	0.1372	0.1151	0.1048	0.1668	0.1310
	3	0.1417	0.1162	0.1015	0.1604	0.1300
C (water–cement ratio)	1	0.1233	0.1437	0.1811	0.0999	0.1370
	2	0.1208	0.1389	0.1697	0.0906	0.1300
	3	0.1153	0.1198	0.1621	0.0776	0.1187

## Data Availability

The original contributions presented in the study are included in the article, further inquiries can be directed to the corresponding authors.
